# Generation of Anti-Idiotype scFv for Pharmacokinetic Measurement in Lymphoma Patients Treated with Chimera Anti-CD22 Antibody SM03

**DOI:** 10.1371/journal.pone.0096697

**Published:** 2014-05-09

**Authors:** Qi Zhao, Pui-Fan Wong, Susanna S. T. Lee, Shui-On Leung, Wing-Tai Cheung, Jun-Zhi Wang

**Affiliations:** 1 School of Biomedical Sciences, The Chinese University of Hong Kong, Shatin, Hong Kong, China; 2 Institute of Biomedical Sciences, Fudan University, Shanghai, China; 3 School of Life Sciences, The Chinese University of Hong Kong, Shatin, Hong Kong, China; 4 National Institutes for Food and Drug Control, Beijing, China; University of Windsor, Canada

## Abstract

Pre-clinical and clinical studies of therapeutic antibodies require highly specific reagents to examine their immune responses, bio-distributions, immunogenicity, and pharmacodynamics in patients. Selective antigen-mimicking anti-idiotype antibody facilitates the assessment of therapeutic antibody in the detection, quantitation and characterization of antibody immune responses. Using mouse specific degenerate primer pairs and splenocytic RNA, we generated an idiotype antibody-immunized phage-displayed scFv library in which an anti-idiotype antibody against the therapeutic chimera anti-CD22 antibody SM03 was isolated. The anti-idiotype scFv recognized the idiotype of anti-CD22 antibody and inhibited binding of SM03 to CD22 on Raji cell surface. The anti-idiotype scFv was subsequently classified as Ab2γ type. Moreover, our results also demonstrated firstly that the anti-idiotype scFv could be used for pharmacokinetic measurement of circulating residual antibody in lymphoma patients treated with chimera anti-CD22 monoclonal antibody SM03. Of important, the present approach could be easily adopted to generate anti-idiotype antibodies for therapeutic antibodies targeting membrane proteins, saving the cost and time for producing a soluble antigen.

## Introduction

For the development of therapeutic antibodies that target membrane antigens, it is important that exogenous naïve soluble antigens are made available for use in quality evaluation and pharmacokinetic assessments of the administered antibodies during preclinical and clinical studies [1]. In the event when such a naïve soluble antigen is not available or accessible, the development of a specific anti-idiotype (anti-Id) antibody could prove handy as a surrogate antigen for the above purposes [2], [3], [4]. Furthermore, the anti-Id antibody can be used as diagnostic reagents for monitoring the pharmacokinetics (PK) of the administered antibody in the circulation of patients. Similarly, it can be used as a positive control for human anti-human antibody (HAHA), human anti-chimeric antibody (HACA) or human anti-murine antibody (HAMA) immune responses to the administered antibody. Monitoring the presence of such immune responses will influence treatment options as such immune responses may affect the clinical outcome in patients [5].

The development of anti-Id antibodies could be laborious and time-consuming, especially employing traditional hybridoma technology [6]. By taking advantage of phage display technology [7], [8], anti-Id single chain Fv (scFv) antibody could be rapidly identified through rounds of panning against idiotype antibody antigen [9], [10]. However, the constraints on folded V domain might render the scFv antibody structurally unstable with a reduced affinity [11], limiting its use in clinical applications. Indeed, no existing evidence supports the use of scFv antibody as surrogate antigen for PK characterization of circulating therapeutic antibody in patients.

SM03 is a chimera anti-CD22 monoclonal antibody (MAb) [12] that is being used in clinical trials for the treatment of non-Hodgkin's lymphoma (NHL) [13]. The antigen is expressed on the surface of matured B cells [14], [15], and upon binding to the antigen, the antibody-antigen complex is rapidly internalized [12], [16]. Since SM03 targets and suppresses matured B cells, the antibody has expanded its indications for the treatment of other autoimmune diseases, such as rheumatoid arthritis (RA) and systemic lupus erythematous (SLE).

To enhance the therapeutic applicability of SM03, a humanized version of SM03 using the technology of framework-patching was also developed [16]. The humanized anti-CD22 antibody SM03 was later renamed as SM06. Both SM03 and SM06 target the same epitope of the human CD22 antigen, with comparable affinity [12], [16]. However, in terms of sequence and structure, SM03 and SM06 share in common only their antigen binding site (ABS) which is formed by their respective complementarity determining region (CDR) sequences.

Exogenous CD22, the natural ligand for SM03 and SM06, is not widely available, making the clinical evaluation of SM03 difficult. In order to develop assay methods for consistent and reliable QC analysis, and for pharmacokinetic evaluation of serum SM03 or its derivatives, an alternative to exogenous CD22 acting as a surrogate antigen is therefore urgently needed.

Here we report the generation of a specific and high affinity anti-Id scFv antibody for the anti-CD22 monoclonal antibody SM03. To bypass the time-consuming and labor-intensive hybridoma preparation, anti-idiotype antibodies were identified from a phage-displayed antibody library which was prepared using specific degenerate primer pairs and splenocytic RNA of mouse immunized with the idiotype anti-CD22 antibody. Moreover, efforts have been made to demonstrate the anti-idiotype scFv antibody acting as a surrogate antigen for membrane protein CD22, and its application in monitoring serum anti-CD22 antibody in lymphoma patients treated with the anti-CD22 antibody.

## Materials and Methods

### Animals and cell lines

The protocol for animal work was approved by the Animal Experimentation Ethics Committee of the Chinese University of Hong Kong (Permit Number: 05/001/ERG). Female BALB/c mice, 6 to 8 weeks old were obtained from the University Laboratory Animal Services Center (CUHK, HK). Mice were housed in a pathogen-free environment with 12 hr dark-light cycle, and allowed to access water and food *ad libitum*.

Human lymphoma cell line, Raji, was purchased from American Type Culture Collection (ATCC, USA). Cells were cultured in RPMI 1640 medium supplemented with 10 % fetal bovine serum (Invitrogen, USA).

### Antibodies

The chimera anti-CD22 MAb (SM03), humanized (framework-patched) anti-CD22 MAb (SM06), humanized (framework-patched) anti-CD20 MAb (SM09) and chimera anti-TNFα MAb (N009) were generated and purified by SinoMab Bioscience Limited (Hong Kong). Murine anti-human CD22 MAb (RFB4) was purchased from Ancell (MN, USA). Horseradish peroxidase (HRP)- and fluorescein isothiocyanate (FITC)-conjugated goat anti-human IgG Fc antibodies were purchased from Jackson ImmunoResearch (PA, USA). Mouse anti-His and anti-M13 antibodies were obtained from GE Healthcare (USA).

### Immunization

The BALB/c mouse was immunized intraperitoneally with 100 µg of SM03 in complete Freund's adjuvant (Sigma-Aldrich, St. Lous, MO) on day 0 and the same amount in incomplete Freund's adjuvant (Sigma-Aldrich) at 2 and 5 weeks following the first immunization. Four days after final immunization, the spleen and serum of the immunized mouse were harvested.

### Construction of phage-displayed mouse scFv library

Total RNA was isolated from mouse splenocytes using Trizol reagent (Invitrogen, Carlsbad, CA) and 10∼15 µg of total RNA was used to prepare first strand cDNA [17]. The cDNA fragments encoding immunoglobulin variable regions were amplified by PCR using specific degenerate primer pairs as described previously [18] with minor modifications. Briefly, PCR amplification of Ig V_H_ and V_Lκ_ was separately carried out in 50 µL reaction volume with 1X PCR buffer (100 mM Tris-HCl; pH 8.3, 500 mM KCl) containing 1.5 mM MgCl_2_, 0.2 mM dNTP, 0.05 U/µL Taq polymerase, 5 µL of cDNA. For V_H_ amplification, 0.3 µM forward primer FH2 (5′-gAggTgMWgcTKVWgSAgTcTggA-3′) and 1.5 µM reverse primer of RH2 (5′-gAcDgTgASHRDRgTBccTKSRccccA-3′) were used. For V_Lκ_ amplification, 0.5 µM forward primer FK12 (5′- gAHRTYgTKMTSAcMcARWcTMcA-3′) and 1.125 µM reverse primer of RK12 (5′-KATYTccARYYTKgTSccHBcDccgAA-3′) were used. For the primer nucleotide sequence, the non-standard bases are: R = A+G; Y = C+T; M = A+C; K = G+T; S = G+C; W = A+T; H = A+C+T; B = G+C+T; D = A+G+T; V = A+G+C. After pre-denaturation at 94°C for 2 min, samples were subjected to 30 cycles of denaturation at 94°C for 30 s, annealing at 58°C for 30 s, and extension at 72°C for 30s, followed by a post-extension at 72°C for 5 min. The PCR products were stored at 4°C until use.

To purify the heavy and light chain variable region gene fragments, the PCR products of 8–10 reactions were pooled and separated in agarose gel. The Ig amplicons were electro-eluted, purified by phenol chloroform extraction and then precipitated by ethanol. To assemble the purified Ig V_H_ and V_Lκ_ DNA fragments into scFv, linkers containing *Sfi* or *Not I* restriction site were added to the purified V_H_ or V_Lκ_ DNA fragments by PCR, respectively. Then the linker-added V_H_ and V_Lκ_ DNA fragments were joined together using over-lapping extension PCR. The nucleotide sequences of those linkers and primer pairs, and the PCR protocols were detailed in our previous publication [18]. Phage-displayed scFv library was constructed using a recombinant phage antibody system following the manufacturer's specifications (GE Healthcare).

### Selection of anti-idiotype scFvs

Phage propagation, either as filamentous phage or in the form of phage-displayed scFv library, was performed as described previously [18]. To select anti-idiotype antibody, the phage-displayed library was separately panned against chimera anti-CD22 SM03 or mouse anti-human CD22 RFB4 on 24-well microplates (IWAKI, Japan). Briefly, ∼10^12^ phages were biopanned against the chimeric (SM03) or murine (RFB4) anti-CD22 MAb in wells coated with 10 µg/well of the respective MAbs in carbonate coating buffer (15 mM Na_2_CO_3_, 35 mM NaHCO_3_, pH 9.6). After incubation at room temperature for 2 h with gentle shaking, bound scFv-phages were eluted with 100 µL of 0.1 M glycine–HCl, pH 2.2. The eluted phages were further incubated at room temperature for 10 min before they were neutralized with 10 µL of 1 M Tris–HCl, pH 8.0. The panning process was repeated four times (four rounds of selection). For each round of panning, the input and output phage titers were determined. An enrichment factor (EF) was calculated using the formula: EF =  (Output/Input)_n_/(Output/Input)_First round_].

### Binding specificity of selected phage clones

The binding specificities of selected phages were assessed against chimeric SM03, murine RFB4, chimeric anti-TNFα MAb (negative control), and humanized anti-CD20 SM09 (negative control) by phage ELISA. Individual colonies surviving the fourth round of selection were randomly picked and inoculated into 2×YT medium (500 µL/well) containing 2% glucose and 100 µg/mL of ampicillin in a 96-well cluster plate (Corning, Tewksbury, MA) and incubated at 37°C overnight with shaking at 250 rpm. Replicas of the master plates were prepared by adding 20 µL of the overnight culture from the master plates to 1 mL/well of 2×YT medium supplemented with 2% glucose and 100 µg/mL of ampicillin in a fresh 96-well cluster plate. After growing at 37°C for 1.5 h with shaking at 250 rpm, ∼10^9^ pfu of M13KO7 helper phage was added to each well of the cluster plates. Then, the 96-well cluster plates were incubated for 1 h at 37°C with shaking at 250 rpm to allow helper phage infection. The infected bacterial cells were collected by centrifugation at 3000×g for 10 min at room temperature. The medium was quickly discarded and the bacteria in each well were resuspended in 1 mL of 2×YT medium containing 100 µg/mL ampicillin and 50 µg/mL of kanamycin. The bacterial culture was incubated overnight at 37°C with shaking at 250 rpm, and culture medium containing the phages was collected and directly tested for antigen binding by phage-ELISA as described previously [18]. Briefly, phage-ELISA was carried out in a 96-well ELISA plate (Nunc, Denmark). The wells were coated with target antibodies (0.1 µg/well) in carbonate coating buffer, pH 9.6, washed once with borate buffer (26 mM Na_2_B_4_O_7_, 100 mM H_3_BO_3_, 0.1% BSA, 100 mM NaCl, 3 mM KCl and 0.5% Tween-20, pH 8.0), and nonspecific binding sites were blocked by incubating with the boarte buffer at 37°C for 1 h before phages were added. Bound phages were detected by incubating with HRP-conjugated mouse anti-M13 antibody. Activity of HRP was quantified using *o*-phenylenediamine (OPD) (Sigma-Aldrich) as substrate; and absorbance at 450 nm was determined with aµQuant micro-plate reader (Bio-Tek, USA).

### Expression and purification of anti-Id scFv

The phage clone Hc5 was selected for further analysis. The DNA fragment encoding the Hc5 scFv was subcloned into pET-His vector. *E.coli* (BL21 (DE3) plysS) transformed with the pET/Hc5 expression vector was induced with 1 mM isopropyl β-D-thiogalactoside (USB, USA), and the inclusion body containing the his-tagged Hc5 scFv was purified with a Ni-NTA agarose column (Invitrogen) and refolded as described previously [19].

### Affinity determination of anti-Id scFv

Binding affinity of Hc5 scFv was determined using BIAcore 3000 system (Biacore, Inc., Sweden) with carboxymethylated dextran-coated CM5 sensor chips and HBS EP running buffer (10 mM Hepes pH 7.4, 150 mM NaCl, 3 mM EDTA, 0.005% P20 surfactant) following standard procedures. Briefly, chimeric SM03 was immobilized on the CM5 sensor chip. The chip surface was firstly activated with 200 mM EDC and 50 mM NHS. Chimeric SM03 in 10 mM sodium acetate buffer (pH 4.5) was injected at a flow rate of 10 µL/min until the immobilization level reached 2000 resonance units (RU). The residual carboxyl groups were subsequently blocked by 1 M ethanolamine (pH 8.5). A control surface was likewise prepared but with chimeric anti-TNFα MAb (N009). The kinetic analysis was performed by injecting serially diluted Hc5 scFv in running buffer over the chimeric SM03 and control surfaces for 3 min at a flow rate of 10 µL/min. The dissociation was studied by washing with running buffer for 15 min. The chip surface was then regenerated by injection with 50 mM NaOH for 30 s. Kinetic parameters including *k*_on_ (association rate constant) and *k*_off_ (dissociation rate constant) were estimated using the BIA evaluation 3.1 software (BIAcore, Inc.). The specific binding curves were normalized by subtracting the control curve.

### Competitive Flow Cytometry Assay

A competitive flow cytometry assay was carried out using Hc5 scFv as a competitor to inhibit the binding of chimeric SM03 to native CD22 antigen on Raji cells which are human Burkitt's lymphoma. The Hc5 scFv was serially diluted with PBS containing 1% BSA in the presence of fixed amount of chimeric SM03 (1 µg/mL). The antibody mixture at each dilution was incubated with 2×10^6^ Raji cells on ice for 1 h. Cells were washed twice with PBS, and the binding of SM03 on Raji cells in the presence of varying concentration of competing Hc5 scFv was determined by adding 100X diluted FITC-conjugated goat anti-human Fc antibody (Jackson ImmunoResearch, West Grove, PA) at 37°C for 1 h. Fluorescent intensity of cells was examined using flow cytometer FACScanto (Becton Dickinson, Franklin Lakes, NJ). Percentage inhibition was calculated according to the formula: 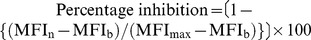, where MFI is mean fluorescence intensity, n represents samples, b represents blank, max represents binding of chimeric SM03 in the absence of Hc5 scFv.

### Capture ELISA Assay

In order to evaluate the capture efficiency of Hc5 scFv to target MAb, ELISA plates were coated with 100 µL of purified Hc5 scFv at a concentration of 10 µg/mL in carbonate coating buffer; pH 9.6. The ELISA plate was washed with borate washing buffer, and nonspecific binding sites were blocked by incubating with the same buffer at 37°C for 1 h. Chimeric SM03 and control chimeric anti-TNFα MAb at different dilutions were added into the wells of the coated plate and incubated for 1 h at 37°C. After three washes, the amount of MAb bound was determined by adding HRP-conjugated anti-human Fc antibody (Jackson ImmunoResearch) at 37°C for 1 h. Activity of HRP was determined with OPD as substrate, and absorbance at 450 nm was measured using µQuant micro-plate reader (Bio-Tek). To evaluate the amount of residual antibody in serum using capture ELISA assay, serum samples from patients treated with SM03 instead of MAb were added into Hc5 scFv-coated wells of ELISA plates.

### Phase I Clinical trial of SM03

The study design and drug administration were approved by the China Food and Drug Administration (CFDA), and detailed in previous publication [13]. The phase I study was conducted at Sun Yat-Sen University Cancer Center, and all subjects gave written informed consent. Blood samples of SM03-treated patients were collected by the clinical team in accordance with the ethical principles of the Declaration of Helsinki.

## Results

### Construction of phage-displayed scFv library from mice immunized with chimera anti-CD22 antibody

**Figure 1:**
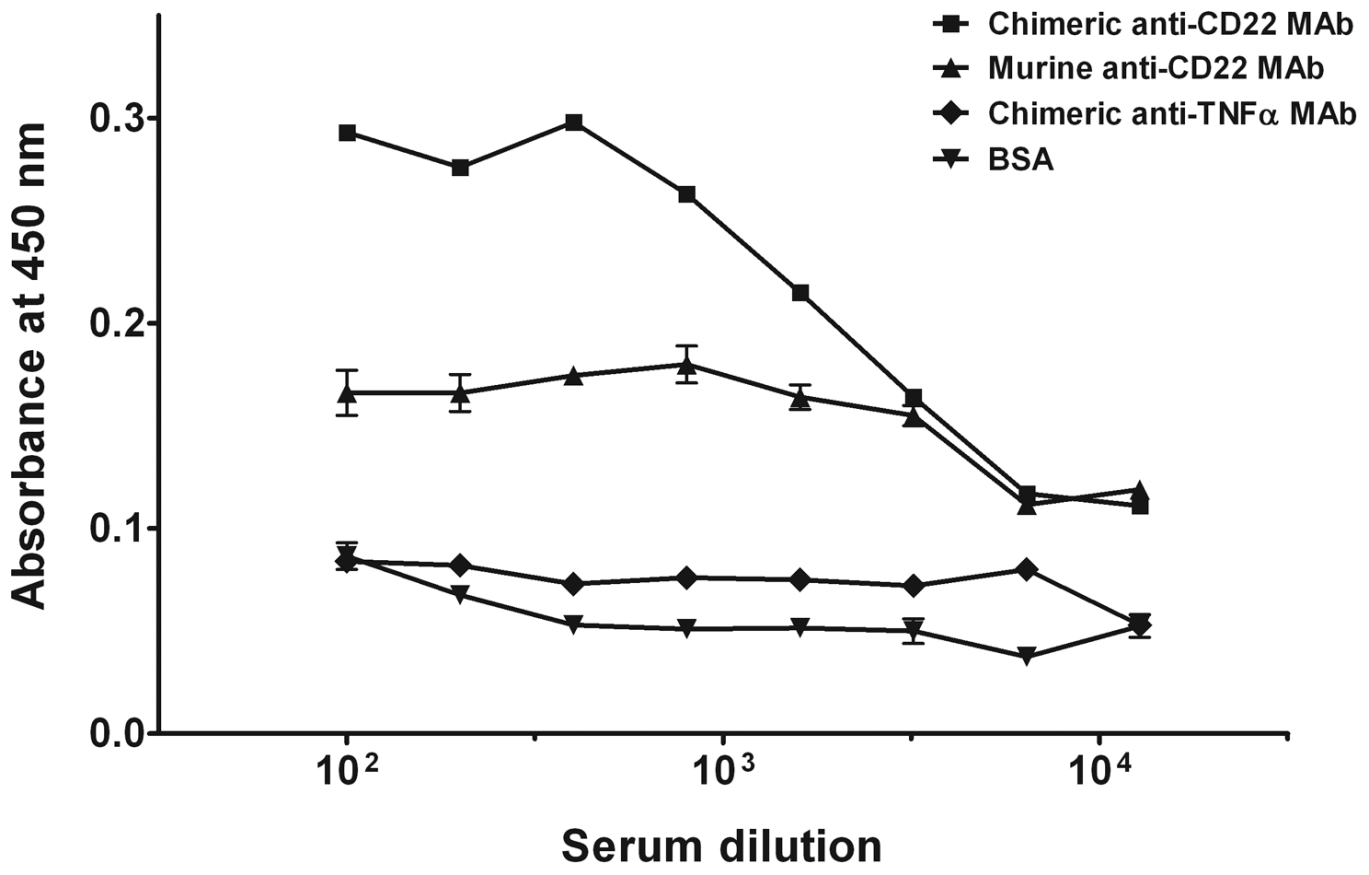
Serum titer evaluation of idiotype antibody-immunized mouse. The BALB/c mouse was immunized with anti-CD22 SM03 as described in Methods. Serum was collected at the fourth day after the second boost dose, and serum titer was evaluated by ELISA. Antigens (0.1 µg/well) that were coated onto the ELISA plate are chimeric anti-CD22 MAb (▪), murine anti-CD22 MAb (▴), chimeric anti-TNFα MAb (⧫), and BSA (▾). Results are expressed as means ± SEM of duplicates. Data shown is a representative of three separate experiments with similar results.

To determine the presence of anti-Id antibody, sera from immunized mice were firstly screened for immunoreactivity against chimeric and murine anti-CD22 antibodies. The immunized mouse serum neither reacted with the control chimeric anti-TNFα antibody (N009) nor with BSA (Figure 1). By contrast, the mouse serum displayed high reactivity towards both chimeric and murine anti-CD22 antibody (Figure 1). These results suggested the presence of antibody against the variable domain, but not the Fc regions, of the anti-CD22 antibody.

To construct a phage-display library of single chain Fv (scFv), cDNA fragments encoding the immunoglobulin variable domain were prepared and amplified from total splenocytic RNA of mouse that displayed high immunoreactivities toward the immunizing idiotype anti-CD22 antibody. It should be noted that only mouse κ light chains were amplified employing the present PCR protocol and included in the scFv library, since λ light chains represent a small proportion of the murine immunoglobulin V gene repertoire. A phage-displayed scFv library was obtained from 10–15 µg of total splenocytic RNA with an estimated size of larger than 1.35×10^8^ independent recombinants.

### Selection and analysis of anti-idiotype scFv clones

To mitigate the possibility of isolating scFvs that preferentially bind to the human Fc region of the immunizing chimeric anti-CD22 antibody, SM03, murine and chimeric SM03 were separately used for panning. Two aliquots of phage scFv library were subjected to four rounds of panning, and enrichment of anti-CD22 binders was observed, either with chimeric SM03 or with its murine counterpart. Phages were enriched 1500-fold and 200-fold after four rounds of panning against chimeric and murine SM03, respectively (Table 1). Consistent with the results of serum titer evaluation (Figure 1), higher enrichment was observed in panning against chimeric SM03 than that of its murine counterpart.

**Table 1:** Enrichment of phages by four consecutive rounds of panning.

Target Antibody	Selection Round	Input Titer^a^	Output Titer^a^	O/I Ratio	Enrichment Factor^b^
Chimeric	1	3.0×10^13^	6.7×10^4^	2.2×10^−9^	1
	2	8.6×10^12^	3.8×10^5^	4.5×10^−8^	20
	3	1.1×10^13^	6.1×10^5^	5.8×10^−8^	26
	4	6.4×10^12^	2.1×10^7^	3.3×10^−6^	1500
Murine	1	3.0×10^13^	1.0×10^6^	3.3×10^−8^	1
	2	4.2×10^12^	8.5×10^4^	2.0×10^−8^	0.6
	3	9.8×10^12^	2.9×10^6^	2.9×10^−7^	9
	4	4.5×10^12^	3.1×10^7^	7.0×10^−6^	212

a Titers are shown as colony-forming unit (cfu)/ml.

b Enrichment factor is defined as:

[O/I Ratio of selection round N]/[O/I Ratio of the first selection round].

**Figure 2:**
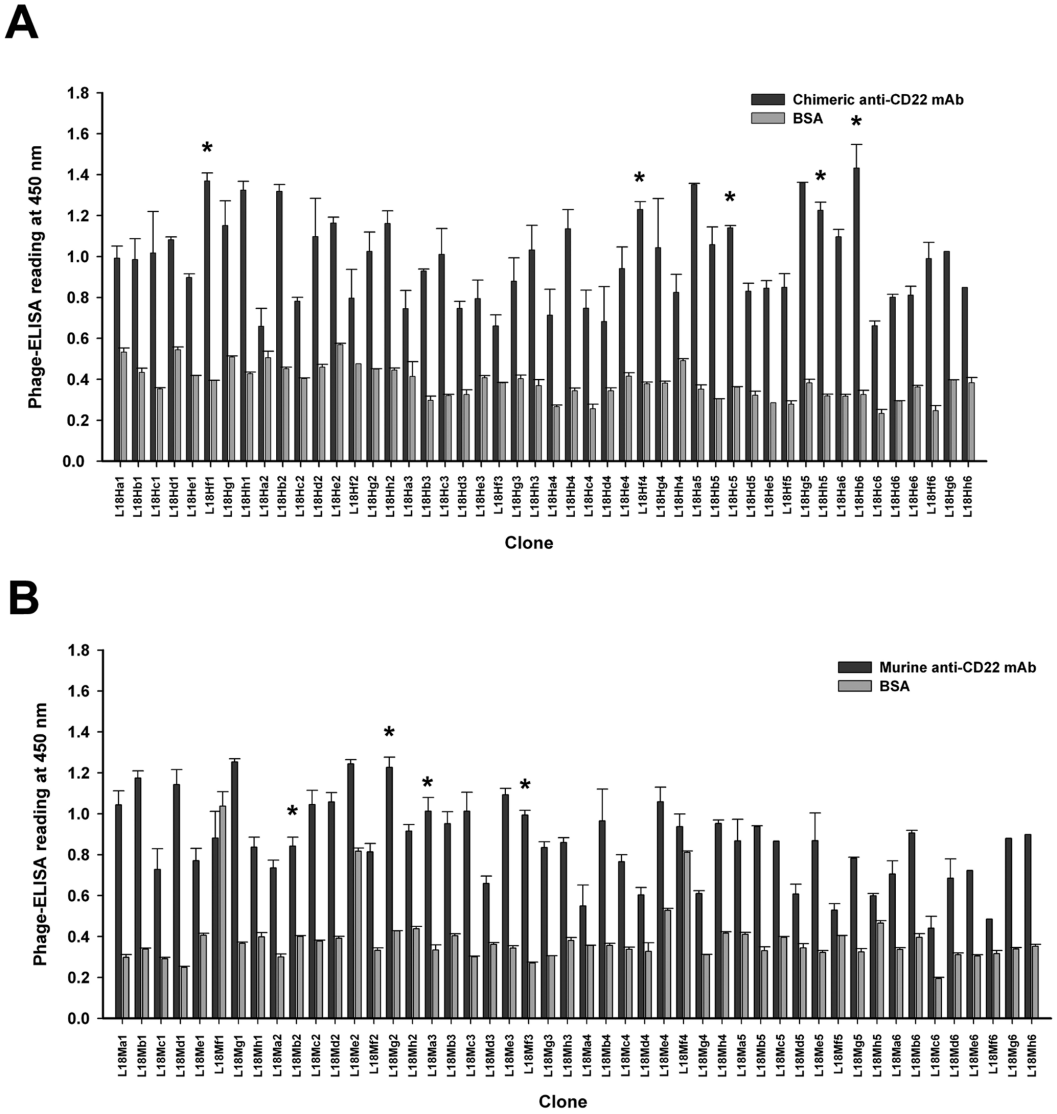
PhageELISA screening of phage clones for reactivity towards idiotype anti-CD22 monoclonal antibody. Individual colonies from the fourth round of biopanning were evaluated in 96-well ELISA plates coated with 0.1 µg/well of MAb or BSA (negative control). Freshly prepared phages were incubated for 1 h against chimeric anti-CD22 MAb (A) or murine anti-CD22 MAb (B). Bound phages were detected with HRP-conjugated anti-M13 as described in Methods. Data shown are means ± SEM of duplicates. The asterisk (*) denotes phage clones that were selected for further evaluation.

To confirm the binding specificity of those selected anti-CD22 binders, individual phage clone was randomly picked and their bindings to their respective panning antigens, chimeric or murine SM03, were evaluated by phage ELISA. As shown in Figure 2, all 48 clones derived from panning against chimeric SM03, and 46 out of 48 clones derived from panning against its murine counterpart, showed strong binding to the respective antigens, but not to the BSA control.

Subsequently, DNA sequences of the randomly picked phages were determined. All of the 20 randomly selected phage clones (10 from each panning) were found to encode scFvs that shared a high homology in DNA sequences, and therefore the translated protein sequences, especially at the CDR.

### Binding specificity of anti-idiotype scFv clones

**Figure 3:**
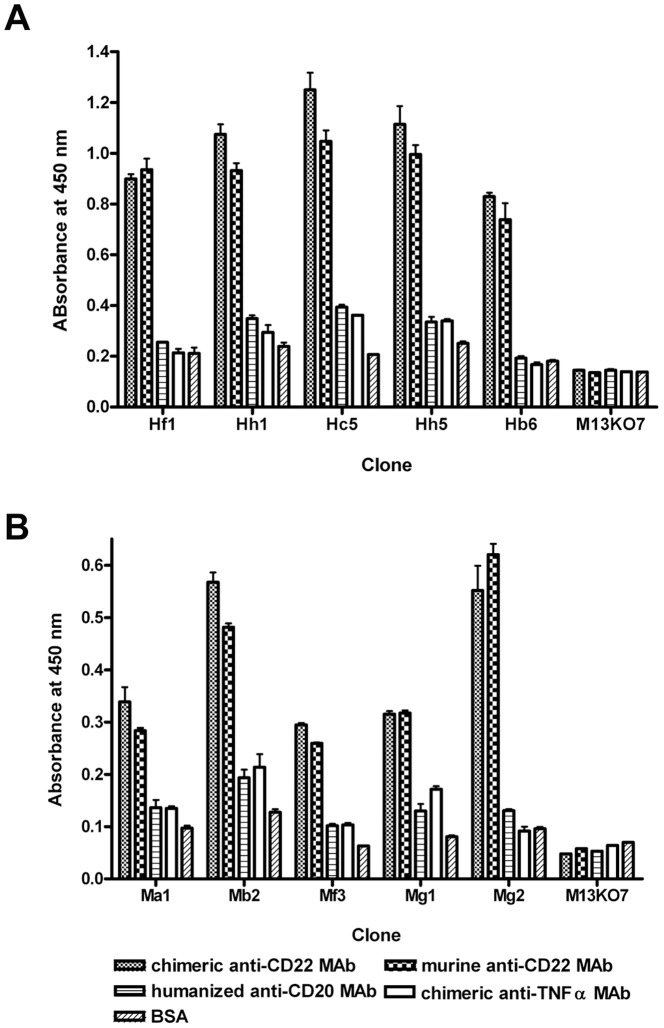
Binding specificity of selected scFv phage clones. Binding specificity of selected phage clones derived from panning against chimeric (A) or murine (B) anti-CD22 MAb were examined by phageELISA as described in Methods. Freshly prepared phages were incubated with 0.1 µg/well of chimeric anti-CD22 MAb (column bars with diffuse dots pattern), murine anti-CD22 MAb (column bars with chessboard pattern), humanized anti-CD20 MAb (column bars with horizontal lines pattern), chimeric anti-TNFα MAb (open column bars), or BSA (column bars with diagonal lines pattern) for 1 h, and bound phages were detected with HRP-conjugated anti-M13. Results are expressed as means ± SEM of duplicates. Data shown is a representative of three separate experiments with similar results.

To examine whether the enriched anti-CD22 binders were anti-idiotypic, five phage clones were selected from each panning (murine *vs* chimeric SM03), and the clones were evaluated for its ability to bind murine and chimeric anti-CD22 antibody by Phage ELISA. While all phage clones tested showed strong binding to both murine and chimeric SM03, no significant binding was detected against the control humanized anti-CD20 (SM09) and chimeric anti-TNFα (N009) antibodies (Figure 3). The only sequence/structure shared by both murine and chimeric SM03 were the variable regions; the results suggested that these scFv phage clones specifically recognized the variable region, but not the constant regions, of the anti-CD22 antibody SM03.

### Expression and purification of anti-idiotype scFv

**Figure 4:**
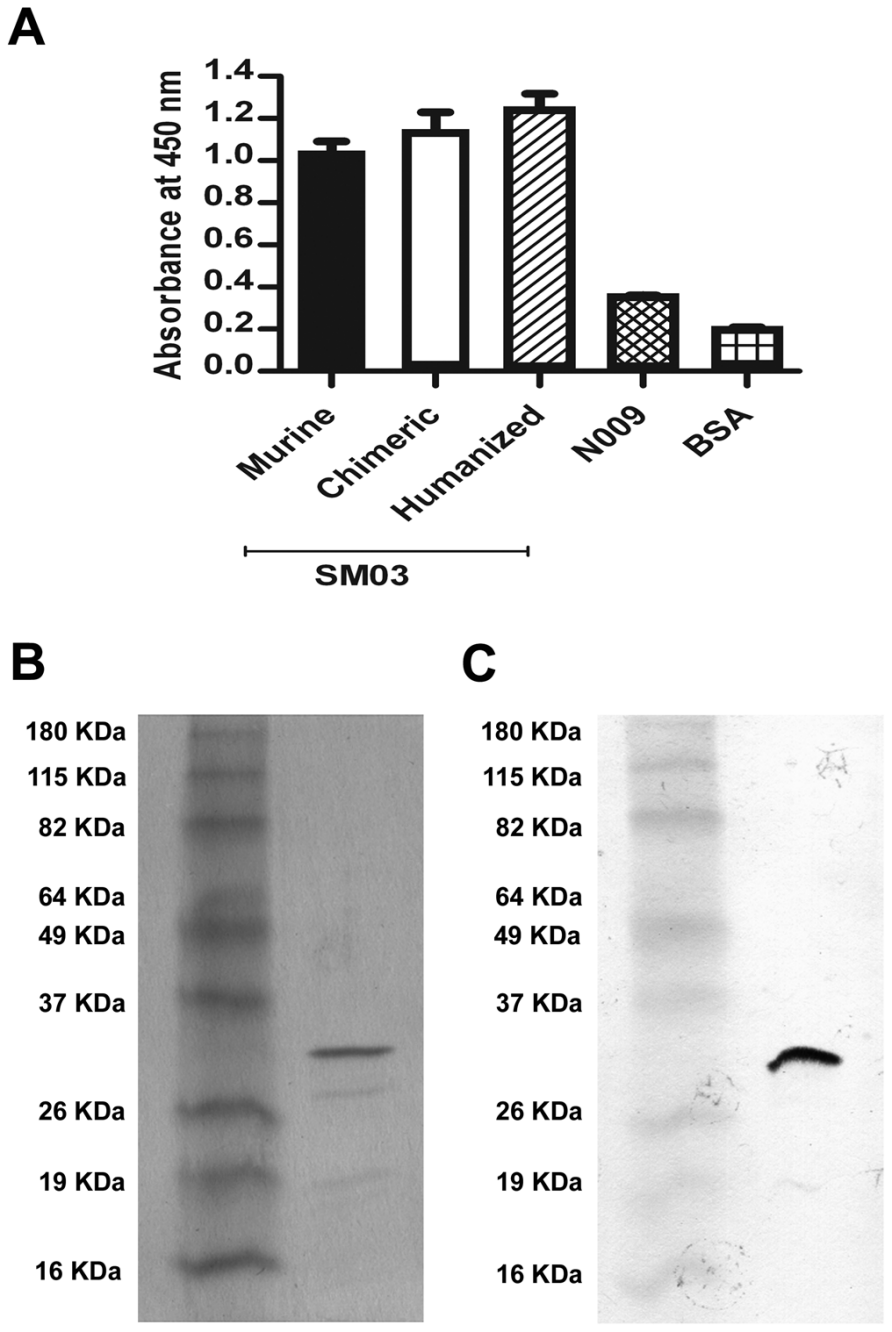
Characterization of anti-idiotype phage clone Hc5. (A) Idiotype binding of phage clone Hc5 was confirmed by its specific binding to immobilized chimera, murine and humanized anti-CD22 (SM03) in phageELISA, but not to negative controls of anti-TNFα (N009) and BSA. Results are expressed as means ± SEM of duplicates. Expression of anti-Id scFv Hc5 was analyzed by SDS-PAGE (B) and western blotting (C). Bacterial expressed and purified His_6_-tagged scFv Hc5 was electrophoresed in 12% acrylamide gel and stained with Coomassie blue. The ScFv Hc5 was transferred onto nitrocellulose membranes and detected with a mouse anti-His_6_ antibody as described in Methods.

The phage clone Hc5 exhibited the highest binding to both murine and chimeric SM03, and was therefore picked for further analyses. As shown in Figure 4a, the Hc5 phage showed significant and comparable binding not just against murine and chimeric SM03, but also against SM06, the humanized version of SM03; bindings to BSA and chimeric anti-TNFα (N009) antibody were, however, weak. Since the sequence motifs shared by murine, chimeric and humanized SM03 resided only in the complementarity-determining region, it was likely that the Hc5 phage, and therefore the expressed scFv borne within the phage, targeted specifically at the antigen binding site of SM03. This result suggested that the binding motif in the Hc5 phage was anti-idiotypic.

Further characterization of the scFv encoded in the Hc5 phage was performed. Briefly, the scFv DNA fragment of phage clone Hc5 was amplified and cloned into a modified pET bacterial expression vector with a His_6_ tag attached at the C-terminus of the scFv. The His_6_-tagged single chain antibody was expressed in *E.coli* BL21 (DE3) as inclusion bodies after IPTG induction, and the solubilized and refolded scFv was purified in a Ni-NTA agarose column. The purified Hc5 scFv appeared as a single band with an estimated molecular weight of ∼30 kDa when subjected to SDS-PAGE (Figure 4b) and western blot (Figure 4c) analyses, confirming the scFv protein being monomeric and of high purity.

### The scFv derived from clone Hc5 was a high affinity anti-idiotype antibody

**Figure 5:**
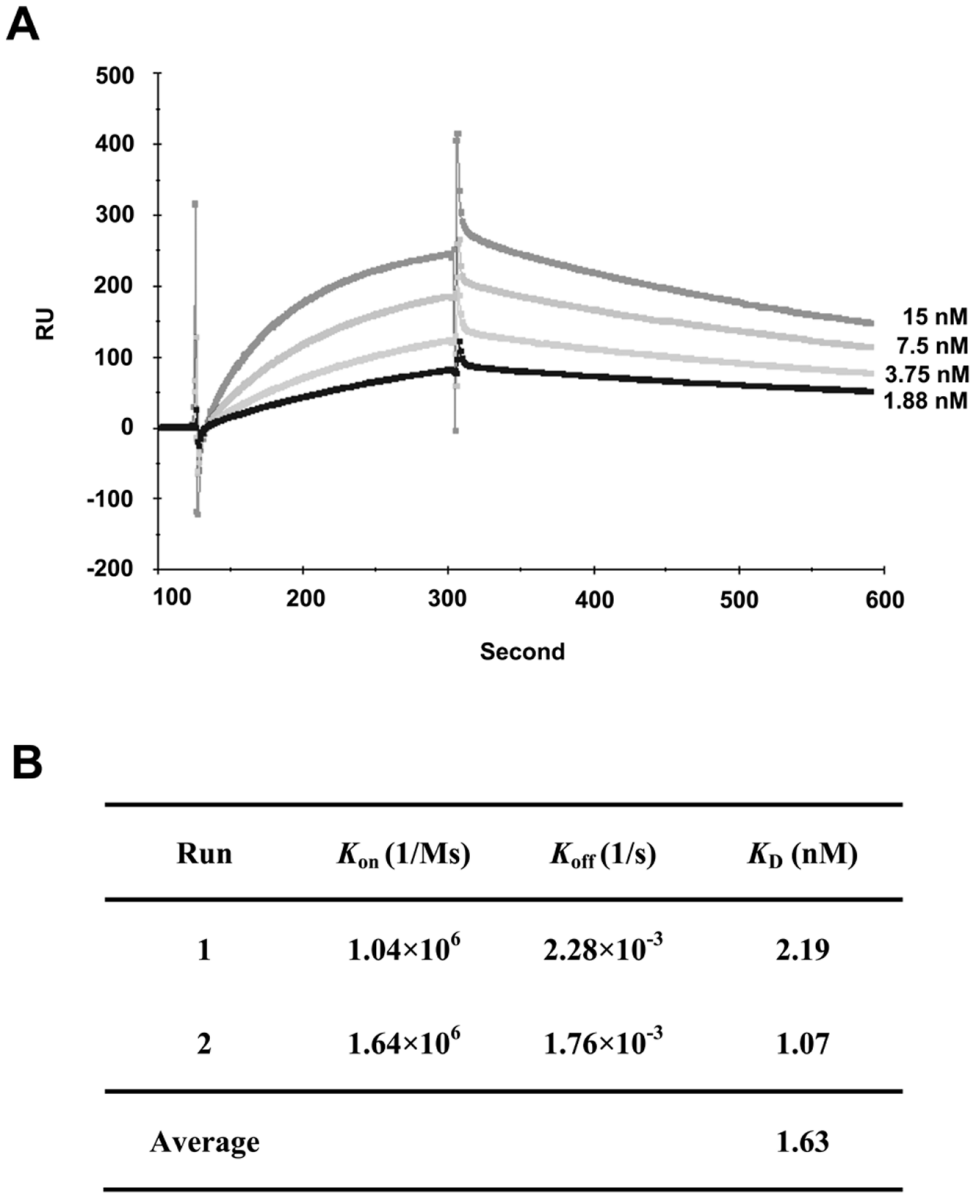
Affinity determination of anti-idiotype scFv Hc5 by surface plasmon resonance. The idiotype chimeric anti-CD22 MAb was immobilized onto Biacore CM5 chip and the anti-Id scFv Hc5 was then applied to determine the binding affinity as described in Methods. (A) Kinetic binding of anti-Id scFv Hc5 was measured by surface plasmon resonance using the BIAcore 3000 system. Numbers in the right margins indicate the concentrations of anti-Id scFv used for each curve. Data shown is a representative of two separate experiments with similar results. (B) The association rate (*k*_on_), dissociation rate (*k*_off_) and affinity constant (*K*_D_) were derived using BIAevaluation software from two separate experiments.

Purified anti-idiotypic Hc5 scFv was subjected to BIAcore analysis for the evaluation of its binding kinetics. The chimeric anti-CD22 SM03 and a control chimeric anti-TNFα N009 monoclonal antibodies were chemically coupled onto a CM5 sensor chip separately. The Hc5 scFvs at various concentrations were then allowed to flow through the chimeric SM03 or the control N009 antibody-conjugated surface of the CM5 chips. The association and dissociation of Hc5 scFvs were being monitored by surface plasmon resonance (Figure 5a). Since both the control chimeric anti-TNFα antibody and chimeric SM03 contained identical Fc regions, the relative binding affinity of Hc5 scFv towards the variable region of chimeric SM03 was obtained by subtracting the value obtained in the anti-TNFα-coated surface with that of the SM03-coated surface. It is of interest to note that the association parameter *k*_on_ was about 9 orders in magnitude larger than that of the dissociation parameter *k*_off,_ indicating the Hc5 scFv was fast in association but relatively slow in dissociation (Figure 5b). The resulting mean *K*_D_ for Hc5 scFv was 1.6 nM which was comparable with that of the anti-idiotype monoclonal antibodies developed against a chimeric anti-CD20 (0.6 nM) [2] and an anti-IL-6 (0.3 to 6.5 nM) [7].

### ScFv Hc5 bound specifically at the idiotope of anti-CD22 SM03

**Figure 6:**
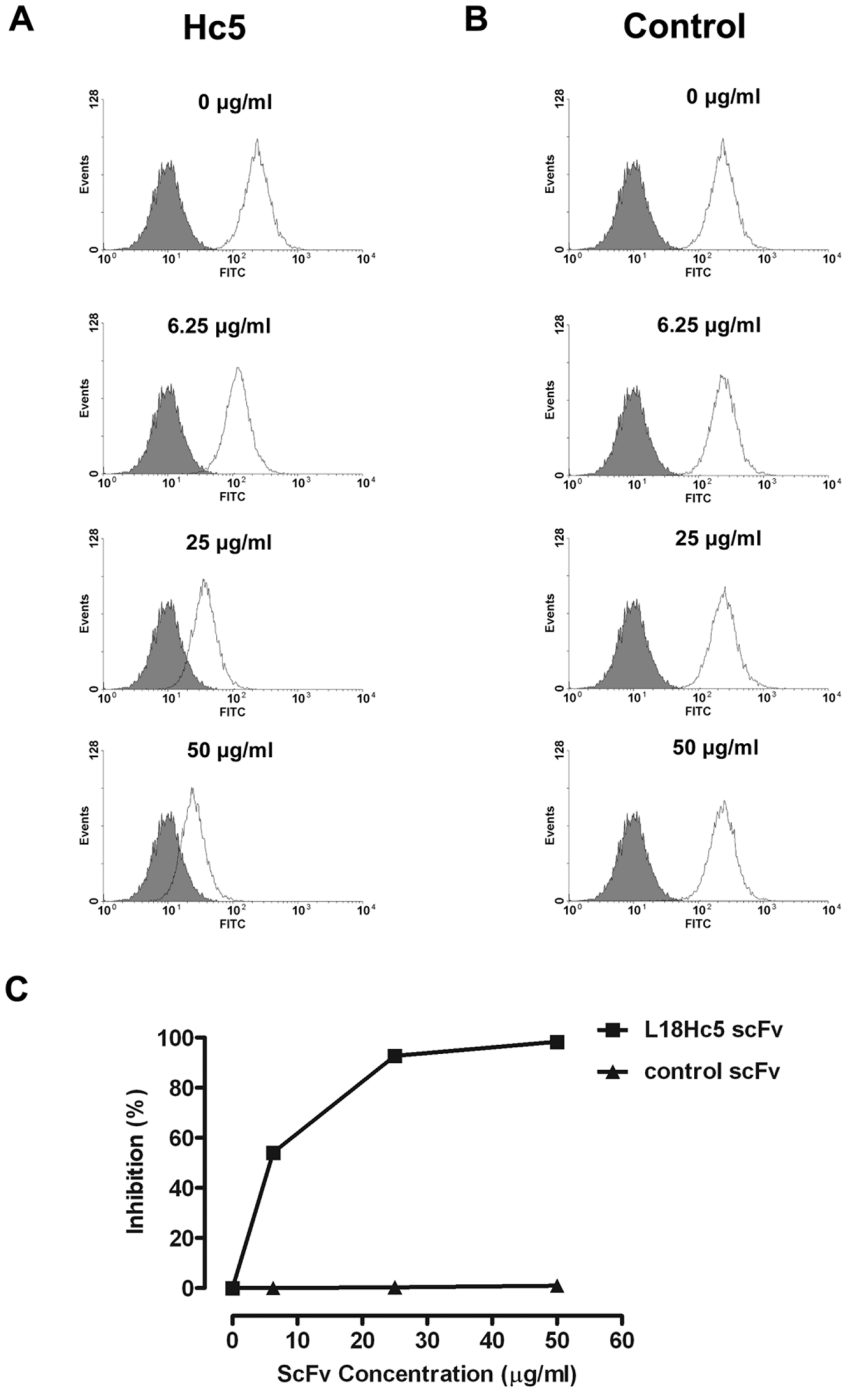
Inhibition of chimeric anti-CD22 MAb binding to CD22 on Raji cells by anti-Id scFv Hc5. Chimeric anti-CD22 MAb at 1 µg/mL was pre-incubated with anti-Id scFv Hc5 or control scFv for 30 min at room temperature. Then the mixtures were incubated with Raji cells (2×10^6^) for 1 h on ice. Following incubation with secondary FITC-conjugated goat anti-human Fc antibody for 1 h on ice, fluorescent intensity of cells was determined by flow cytometry. (A) The histograms showing the distribution of fluorescent intensity of cells that incubated with antibody mixture of SM03 and anti-Id scFv Hc5 (transparent) or blank (solid) at the indicated concentrations. (B) The histograms show the distribution of fluorescent intensity of cells that incubated with antibody mixture of SM03 and a control scFv (transparent) or blank (solid) at the indicated concentrations. (C) The percentage inhibition of anti-CD22 binding by anti-Id scFv Hc5 (▪) and control scFv (▴) with data derived from flow histogram of panels (A) and (B). Data shown is a representative of two separate experiments with similar results.

The SM03 antibody binds to CD22 antigen that expresses on the surface of Burkitt's lymphoma cell lines such as Raji [12]. It was predicted that the Hc5 scFv would block the binding of SM03 onto Raji cells. A competitive flow cytometry assay was performed in which the binding of SM03 onto Raji cells was suppressed by the presence of increasing concentrations of purified Hc5 scFv in a dose-dependent manner (Figure 6a and 6c). No binding inhibition was observed with a control scFv L17E4d which was specifically against a different cell surface protein [20] (Figure 6b and 6c). These results suggested that Hc5 scFv bound to the idiotype of SM03 antibody, and thereby blocked the CD22 binding of the SM03 antibody.

**Figure 7:**
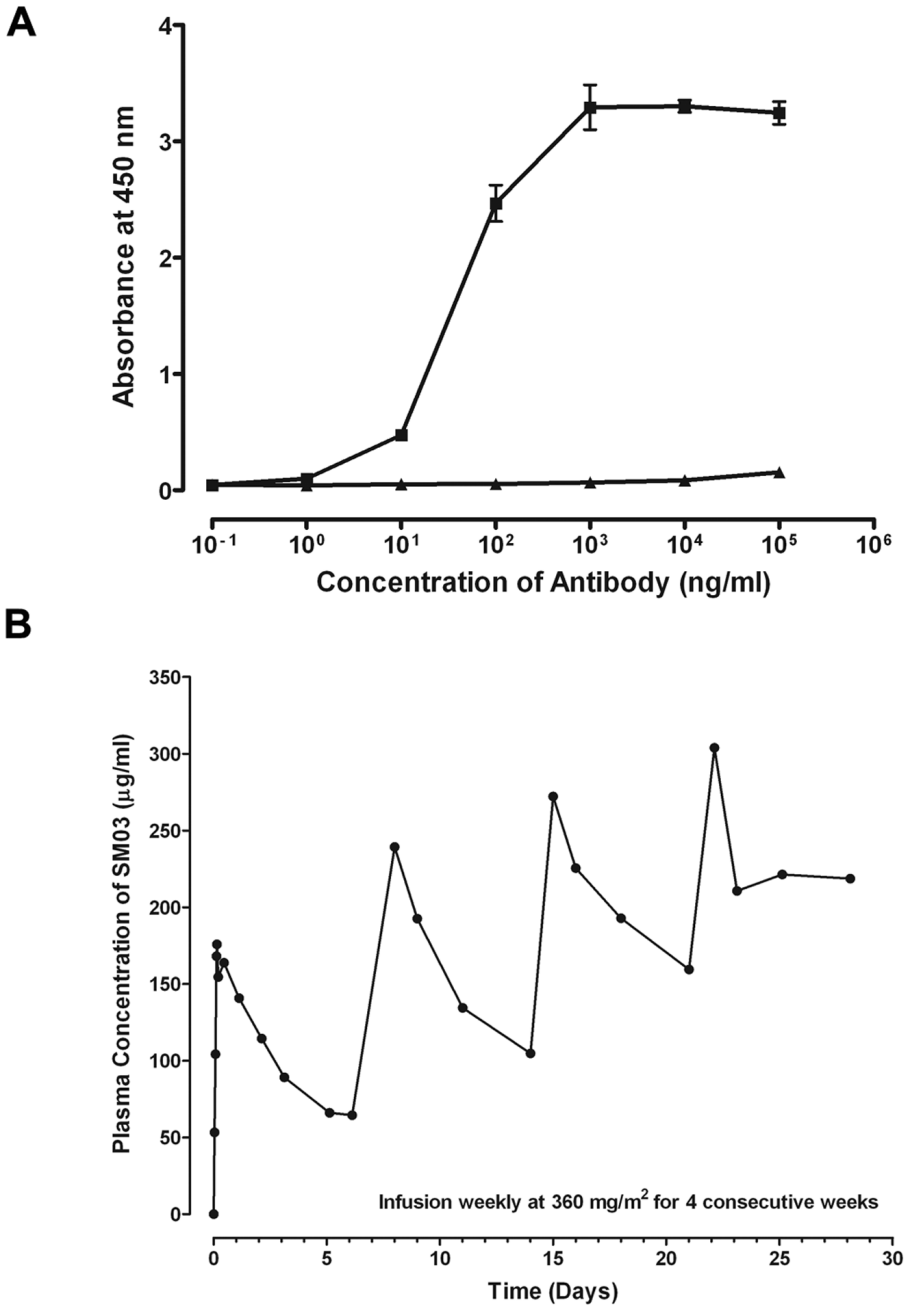
Pharmacokinetic measurement of chimeric anti-CD22 SM03 by capture ELISA. The anti-Id scFv Hc5 was coated onto 96-well ELISA plate at 10 µg/mL, and then incubated at 37°C for 1 h with (A) various amounts of chimeric anti-CD22 SM03 (▪) or control chimeric anti-TNFα N009 (▴) to establish a standard calibration curve; (B) blood samples of a lymphoma patient treated with weekly infusion of 360 mg/m^2^ of chimeric anti-CD22 SM03 to determine residual SM03 in plasma as described in Methods. The captured SM03 antibodies were then detected by incubating with secondary HRP-conjugated goat anti-human IgG Fc antibody. Data shown is a representative showing the typical pharmacodynamic measurement of circulating SM03 in patients undertook the Phase I clinical trial.

To determine whether Hc5 scFv was selective and sensitive in capturing chimeric SM03, a capture ELISA binding assay was established in which the purified Hc5 scFv was firstly immobilized onto the wells of an ELISA microtiter plate, then varying concentrations of chimeric SM03 or control anti-TNFα N009 were added. As shown in Figure 7a, binding of chimeric SM03, but not the control antibody N009, to the immobilized Hc5 scFv was dose-dependent, with an estimated EC_50_ value of 43.19±1.0 ng/mL (n = 3).

### Use of anti-Id scFv Hc5 for pharmacokinetic measurement in Chinese non-Hodgkin lymphoma patients treated with chimeric SM03

Chimeric anti-CD22 SM03 was subjected to Phase I dose-escalation clinical studies in years 2007 to 2008 to evaluate its safety, pharmacokinetic properties and biological effect in Chinese non-Hodgkin lymphoma patients [13]. Using a capture assay for pharmacokinetic characterization, immobilized anti-idiotype scFv Hc5 (then named as LRID03) was used as a surrogate ligand for CD22 to determine the serum levels of residual SM03 in lymphoma patients treated with the antibody. The presence of human serum and other proteins did not seem to interfere with the capture of SM03 onto the immobilized Hc5 scFv (data not shown). Patients were treated with the chimeric SM03, once a week, for four consecutive weeks at varying doses. Figure 7b illustrated a typical PK profile of a patient treated with SM03 at a dose of 360 mg/m^2^. The levels of residual SM03 in circulation decreased gradually over the weekly injection cycle, typical of a therapeutic monoclonal antibody. The basal levels of circulating SM03 escalated over the four injection cycles, suggesting that the surface binding epitopes of peripheral B cells were saturated with SM03 at the dose of 360 mg/m^2^. Indeed, pharmacokinetics study of SM03 at the dose of 360 mg/m^2^ indicated a mean C_max_ and a mean AUC_0→t_ value of 196.03 µg/mL and 16.81 mg h/mL, respectively [13].

## Discussion

We showed herein that an anti-idiotype scFv Hc5 could act as a surrogate ligand for membrane protein CD22. In addition, we also demonstrated an efficient strategy for generating anti-idiotype antibodies using an idiotype antibody-immunized phage-displayed antibody library that was prepared with mouse specific degenerate primer pairs. Importantly, the anti-idiotype scFv Hc5 was target-specific with high affinity, and was successfully used for pharmacokinetic analysis of circulating residual antibody in lymphoma patients treated with the idiotype anti-CD22 antibody SM03.

SM03 is a chimeric antibody that targets the B epitope of human CD22 antigen [12], and is currently in different phases of clinical trials in China for the treatment of non-Hodgkin's lymphoma (NHL), and other autoimmune diseases. As one of the requirements for conducting clinical trials, it is imperative that the residual amount of circulating SM03 antibody could be monitored and measured during the period of the clinical studies. Robust bioanalytical pharmacokinetic studies will provide information such as Cmax, Tmax, T_1/2_ and AUC that are related to absorption, distribution, elimination, interactions and possible adverse reactions of the anti-CD22 SM03. These data are used to evaluate antibody exposure and safety as well as to characterize pharmacodyamic relationships. Therefore, accurate measurement of therapeutic antibodies in serum or plasma of patient is an important aspect for clinical PK studies [21].

Determination of the residual antibody could be easily accomplished if exogenous or soluble form of the target antigen is readily available; as the antigen could help establish appropriate assays or test methods not only for quality control during production but also PK evaluation during clinical trials. However, soluble CD22, the target antigen for SM03, is not readily available. It would be costly if soluble CD22 is used as the capture antigen for the detection of serum SM03 and as routine reagent for general quality control. Other groups working on anti-CD22 antibodies circumvented the problem by developing anti-idiotype antibody, a commonly used alternative to naive antigen, as the surrogate antigen for CD22 [22], [23], [24]. However, standard procedure for the development of anti-idiotype antibody can be laborious and time-consuming [22].

Phage display technology, to a great extent, simplifies and shortens the process for hybridoma preparation and screening. However, to succeed in the identification of high affinity antibody, it is essential that a phage-displayed antibody library that mirrors the full antibody responses from the immunized mice could be prepared. Recently we showed a novel method for establishment of a highly diversified phage-displayed antibody library using splenocytic genomic DNA [18]. Employing the same degenerate primer pairs, but with splenocytic RNA, we constructed an antibody library encompassing the Fv repertoires of immunized mouse, and successfully identified scFv antibody fragment that bound specifically and strongly to the anti-CD22 antibody SM03. The identification of the anti-SM03 anti-idiotype antibody was facilitated by the availability of murine, chimeric and humanized versions of SM03. Alternate panning and binding studies with the three different forms of SM03 proved to help eliminate binders that targeted the constant regions or portions of the variable regions formed by the framework sequences.

The scFv clone Hc5 showed specific binding to all three forms of SM03 but not to other control antibodies. Since the CDR is the only sequence or structure shared by all three formats of SM03, it would be safe to assume that Hc5 scFv was an anti-idiotype antibody against SM03. It was further confirmed that Hc5 scFv bound specifically to the antigen binding site of SM03. Moreover, the Hc5 scFv was also shown to inhibit SM03 binding to the natural CD22 ligand on Raji lymphoma cells in a dose dependent manner. It is of interest to note that most of the anti-SM03 phages which showed significant binding to either murine or chimeric SM03 encoded highly homologous scFv sequences, especially at the CDR regions. The result suggests the antibody responses in mice immunized with anti-CD22 were likely dominated by a few B cell clones.

Anti-idiotype antibodies are antibodies that are specific for the unique antigenic determinant, also known as the idiotype, of the immunizing antibody. The immunizing antibody is usually referred to as Ab1, and the anti-idiotype antibody as Ab2. There are three categories of Ab2 antibodies: (1) Ab2α antibodies are those that recognize the idiotype on Ab1 but not the antigen-binding site; (2) Ab2β antibodies recognize epitopes within the antigen binding site of Ab1 with structure resembling the internal image of the nominal antigen; (3) Ab2γ antibodies bind to the epitopes within the ABS without the structural resemblance of an internal image [25].

It is conceivable that Ab2β would draw more attention because they can potentially be used as surrogate antigens for the development of active vaccines against autologous antigens, including those that are immunologically inert, such as tumor-specific or tumor associated antigens [26]. However, other types of Ab2 can also be useful, especially when developing assay methods that would facilitate the production process and clinical evaluation of a potentially therapeutic Ab1. Both direct binding studies and competitive flow cytometry studies confirmed that Hc5 scFv targeted the ABS of SM03, ruling out the possibility that Hc5 being an Ab2α antibody. Anti-sera obtained from mice immunized with Hc5 scFv antibody failed to show anti-CD22 activities (data not shown), offering no definitive proof that the Hc5 scFv antibody was the Ab2β category. Regardless, it remained possible the immunization was not done properly to result in the development of anti-idiotype activities against the Hc5 scFv, and further characterization studies are therefore needed and are underway. Nevertheless, our results supported that Hc5 scFv was specific for the ABS of SM03, and was either an Ab2β or an Ab2γ anti-idiotype antibody.

Consistent with previous report that a reduced binding affinity is generally observed in scFvs due to the constraints on folded V domain [11], the affinity of anti-idiotype scFvs prepared from a synthetic human antibody library [8] and from a hybridoma cell clone [27] was found to be inµM range. For phase I dose-escalation clinical study, anti-CD22 SM03 was administered at a dose range of between 60 to 480 mg/m^2^, once a week for four consecutive weeks; and the residual antibody levels in blood was calculated to be in the nM to low µM range. Hence, in the development of PK assays, it is preferable to have a scFv that targets the antigen binding site of SM03 with affinity in nM range. Enhancement of affinity was noted in antibodies derived from immunized mouse resulting from hypermutation [28]. Instead of performing the time-consuming and labor-intensive hybridoma screening, we took advantage of our previous protocol of retrieving most, if not all, of the mouse Ig genes in one single PCR step with mouse specific degenerate primer pairs [18]. An idiotype antibody-immunized phage-displayed library was generated using splenocytic total RNA. Following simple *in vitro* panning and ELISA screening, high affinity anti-idiotype scFv clones were quickly isolated. Indeed, the anti-Id Hc5 scFv was able to capture circulating SM03 and successfully used to delineate the PK profiles of different lymphoma patients treated with SM03 in a phase I dose-escalation clinical study [13].

Besides being used as surrogate antigen, there are other potential applications that anti-idiotype scFv or its derivatives can prove useful. For example, the Hc5 scFv could be used to develop assay to confirm the ABS identity of antibodies, especially when multiple antibody candidates are being developed in the same facility; or as a positive control for assays examining HACA response. We are also evaluating the possibility of using the Hc5 scFv as a surrogate antigen for monitoring affinity changes, if any, for SM03 or its derivatives, during the production and purification process. This will greatly facilitate the in-process QC as the assay does not rely on the availability of soluble CD22 or the laborious flow cytometry studies.

Comparing to the conventional full format anti-idiotype antibody, anti-idiotype scFv could be more cost-effective and more flexible. An scFv antibody is smaller in size (30 vs 150 kDa), simpler in structure, and easily bacterially expressed and purified in large quantity. However, the constraints on folded V domain might render the scFv antibody structurally unstable and its affinity reduced [11]. Although the Hc5 scFv exhibited high affinity toward the anti-CD22 antibody SM03 at nM range, which was comparable to that of other similar but different anti-idiotype antibody to Epratuzumab, another anti-CD22 antibody that is currently in Phase III clinical trials [29], the refolded scFv tended to become unstable, especially upon storage. To achieve consistency required for general quality control during production, the Hc5 scFv could be converted, by antibody engineering, into a more stable structure, such as IgG molecule. Full immunoglobulin is preferred as the structure is known to be very stable, and the establishment of production cell lines, purifications, and storage are relatively straight-forward and standard in the industry.

Despite ELISA assay in coupled with specific antigen is widely adopted method for pharmacokinetic measurement of circulating residual antibody in patients, the rapid development in technology for plasma protein determination has provided an alternative. Recently, accurate, sensitive and quantitative plasma protein determination has been demonstrated using multiple reaction monitoring (MRM)-mass spectrometry in conjunction with stable isotope labelled target-specific signature peptides [30]. Provided that the amino acid sequence of therapeutic antibody is known, therapeutic antibody-specific signature peptides could be easily custom prepared. In principle, the levels of circulating residual therapeutic antibody could be quickly determined using MRM-mass spectrometry without requiring the availability of a soluble format of target antigen. However, extensive studies are required to establish a rigorous protocol to ensure the consistence and accuracy.

In summary, we reported herein that by employing specific degenerate primer pair to construct an idiotype-immunized phage-displayed scFv library, and by performing parallel screening using murine, chimeric and humanized versions of SM03, we were able to rapidly identify an anti-idiotype scFv phage (Hc5) that bound to the ABS of all three versions of the anti-CD22 SM03. The anti-idiotype Hc5 scFv was expressed in bacteria as inclusion bodies, which were then denatured and refolded as an active scFv. The Hc5 scFv was successfully used to measure circulating residual antibody in lymphoma patients treated with SM03, in a dose-escalating Phase I clinical trial in Sun Yat-Sen Medical Center, Guangzhou, China. The PK profiles of these patients were in agreement with that expected for a chimera antibody.
